# On models of physiologically structured populations and their reduction to ordinary differential equations

**DOI:** 10.1007/s00285-019-01431-7

**Published:** 2019-09-28

**Authors:** Odo Diekmann, Mats Gyllenberg, Johan A. J. Metz

**Affiliations:** 1 Department of Mathematics, University of Utrecht, P.O. Box 80010, 3508 TA Utrecht, The Netherlands; 2 Department of Mathematics and Statistics, University of Helsinki, P.O. Box 68, 00014 Helsinki, Finland; 3 Mathematical Institute and Institute of Biology, Leiden University, 2333 CA Leiden, The Netherlands; 4 Evolution and Ecology Program, International Institute of Applied Systems Analysis, 2361 Laxenburg, Austria

**Keywords:** Renewal equation, Finite dimensional state representation, Cell fission models, 92D25, 93B11

## Abstract

Considering the environmental condition as a given function of time, we formulate a physiologically structured population model as a linear non-autonomous integral equation for the, in general distributed, population level birth rate. We take this renewal equation as the starting point for addressing the following question: When does a physiologically structured population model allow reduction to an ODE without loss of relevant information? We formulate a precise condition for models in which the state of individuals changes deterministically, that is, according to an ODE. Specialising to a one-dimensional individual state, like size, we present various sufficient conditions in terms of individual growth-, death-, and reproduction rates, giving special attention to cell fission into two equal parts and to the catalogue derived in an other paper of ours (submitted). We also show how to derive an ODE system describing the asymptotic large time behaviour of the population when growth, death and reproduction all depend on the environmental condition through a common factor (so for a very strict form of physiological age).

## Introduction

Monod (1942, 1949, 1950; Morin and Monod 1942) presented his now famous ODE-model of batch and continuous cultivation of bacteria feeding on a (growth limiting) substrate:

1.1 $$\begin{aligned} \frac{d}{dt}X(t)= & {} \frac{aS(t)}{b+S(t)}X(t) -DX(t), \end{aligned}$$

1.2 $$\begin{aligned} \frac{d}{dt}S(t)= & {} -\gamma \frac{aS(t)}{b+S(t)}X(t) +D(S_\mathrm{ext} -S(t)), \end{aligned}$$

where *X*(*t*) is the bacterial biomass and *S*(*t*) is the substrate concentration in the growth tank at time *t*. The constant concentration of substrate in an external reservoir is denoted by $S_\mathrm{ext}$ and this is fed into the growth tank at a constant rate *D*. Bacteria and substrate are removed at the same rate, which therefore is called the dilution rate. The reciprocal of $\gamma$ is a conversion factor telling how consumed substrate is converted into bacterial biomass. The case $D=0$ corresponds to batch cultivation.

The model is formulated in terms of bacterial biomass and not in terms of individual cells. The reason is that consumption of substrate results in cell growth and not directly in cell fission. When a cell splits depends on its size, which in turn depends on the history of its substrate consumption, so on the history of the substrate concentration and uptake ability as determined by size. The model should therefore be viewed as a reduction of a more detailed structured population model in the spirit of Tsuchiya et al. (1966), Fredrickson et al. (1967), Gyllenberg (1982) and Diekmann et al. (1984, 1986). The following question arises naturally: Under what conditions on the individual processes, viz. growth, survival and fission, do the Eqs. (1.1) and (1.2) provide a faithful representation of a size-structured cell-based model?

In two recent papers (Diekmann et al. 2018, submitted) we considered the problem of ODE-reducibility of physiologically structured populations from different angles and under different restrictions. In the paper (Diekmann et al. 2018) we focussed on the population level birth rate under the assumption of a finite number of states-at-birth and with individual state (i-state for short) dynamics, for instance, consisting of jumps from one state to another. We gave necessary and sufficient conditions on the kernel of the resulting renewal equation with input for when the birth rate vector can be recovered from the solution of a system of finitely many ODEs. In the paper (Diekmann et al. submitted) we considered deterministic i-state development and, working in the spirit of abstract evolution equations, we gave a complete catalogue, in terms of the individual growth and death rates of models, for which one can find a finite set of population *outputs* that can be recovered from a system of ODEs (see Sect. 6). The restrictions imposed were: the individual state space is one-dimensional and reproduction is part of the output.

The aim of the present paper is

(i) to promote the renewal equation formulation of physiologically structured population models with deterministic i-state development;
(ii) to use that formulation to give a short and easy derivation of the key conditions for ODE reducibility;
(iii) to provide new examples, in particular for reproduction by fission into two equal parts;
(iv) to recall an old example (Diekmann et al. 1983; Metz and Diekmann 1986, I.4.5, II.14) of *asymptotic* ODE reducibility and to show how this fits in with the key condition.

## Physiologically structured population models formulated as delay equations

Traditionally physiologically structured population models are formulated as first order partial differential equations with non-local terms (Metz and Diekmann 1986; Perthame 2007). In the papers (Diekmann et al. 1998, 2001) we developed a constructive method for solving such equations via generation expansion as it is used in the theory of renewal equations (Feller 1971). In the paper (Diekmann et al. 2010) we put the renewal equation centre stage by using the history of the birth rate as state and by defining a dynamical system by updating this history. In the present paper we take the renewal equation as the starting point for modelling physiologically structured populations.

Let $\varOmega \subset {\mathbb {R}}^q$ be the individual state space (i-state space for short), that is, the set of all admissible individual states (i-states). The basic ingredient of a structured population model is

$$\begin{aligned} \lambda _E(t,s)(\xi , \omega ), \end{aligned}$$

which is the rate at which offspring are produced at time *t* in the Borel measurable set $\omega \subset \varOmega$ by an individual who had i-state $\xi \in \varOmega$ at time *s*, given the environmental input *E* as a function of time. Note that Diekmann et al. (1998, 2001) considered the more general class of models that allow for jumps in the cumulative number of offspring produced as a function of the time elapsed, and therefore worked with the Stieltjes integral with respect to the time variable.

More often than not the possible states-at-birth form a subset $\varOmega _b \subset \varOmega$ which is considerably smaller than $\varOmega$. As an extreme, but important, special case we have the situation of only one state-at-birth when $\varOmega _b = \{x_b\}$. We therefore consider $\lambda _E(t,s)(\xi , \cdot )$ as a Borel measure on $\varOmega$ with support in $\varOmega _b$. One of the reasons for working with measures instead of densities is that we can treat the cases of continuously and discretely distributed states-at-birth, as well as more complicated cases, in one go. We denote the $\sigma$-algebra of Borel subsets of a subset *Y* of ${\mathbb {R}}^q$ by ${\mathscr {B}}(Y)$.

Let *b*(*t*) be the population birth rate, that is, *b*(*t*) is a Borel measure on $\varOmega _b$ with the interpretation that $b(t)(\omega )$ is the rate at which offspring with i-state in the set $\omega \in {\mathscr {B}}(\varOmega _b)$ are produced at time *t*. Straightforward book-keeping then yields the following renewal equation with input:

2.1 $$\begin{aligned} b(t)(\omega ) = \int _{-\infty }^t \int _{\varOmega _b} \lambda _E(t,s)(\xi , \omega ) b(s)(d\xi )ds, \quad \omega \in {\mathscr {B}}(\varOmega _b) \end{aligned}$$

(Diekmann et al. 2007). If the birth rate is given on the interval $(-\infty ,s]$ as an initial condition

2.2 $$\begin{aligned} b(t) = \varphi (t), \quad t \le s, \end{aligned}$$

then (2.1) and (2.2) can be written as

2.3 $$\begin{aligned} b(t)(\omega ) = F(t,s)(\omega ) + \int _s^t \int _{\varOmega _b} \lambda _E(t,\tau )(\xi , \omega ) b(\tau )(d\xi )d\tau , \end{aligned}$$

where

2.4 $$\begin{aligned} F(t,s)(\omega ) = \int _{-\infty }^s \int _{\varOmega _b} \lambda _E(t,\tau )(\xi , \omega ) \varphi (\tau )(d\xi )d\tau \end{aligned}$$

is the contribution to the birth rate of the individuals born before the initial time *s*.

Equation (2.1) can be viewed as a linear non-autonomous Volterra integral equation for functions of time with values in the Banach space $M(\varOmega _b)$ of all Borel measures on $\varOmega _b$. Results on existence and uniqueness of such abstract equations are available. The proofs usually mimic the corresponding ones for equations in ${\mathbb {R}}^n$ (Gripenberg et al. 1990) based on Banach’s fixed point theorem and the associated Picard–Lindelöf successive approximations, see e.g. Kvapiš (1967). In our case well-posedness is almost immediate, because the solution can be constructed by way of the *generation expansion* (Diekmann et al. 1998, 2001).

We now introduce a second ingredient $u_E(t,s,\xi )(\omega )$ which is the probability that an individual that had i-state $\xi \in \varOmega$ at time *s* is still alive at time *t* and has i-state in $\omega \in {\mathscr {B}}(\varOmega )$ at that time, given the course of the environment $\tau \mapsto E(\tau ),\,\, \tau \in [s,t]$ (Diekmann et al. 1998, 2001). Once the birth rate *b*(*t*) has been solved from (2.1) the i-state distribution $m(t) \in M(\varOmega )$, often called the population state or p-state, can be obtained from the now explicit formula

2.5 $$\begin{aligned} m(t)(\omega ) = \int _{-\infty }^t \int _{\varOmega _b} u_E(t,s,\xi )(\omega ) b(s)(d\xi )ds, \quad \omega \in {\mathscr {B}}(\varOmega ). \end{aligned}$$

We now specialise to the case of deterministic i-state development which we simply call growth. Let *g*(*x*, *E*) be the individual growth rate of an individual with i-state *x* when the environmental condition is *E*. Then the i-state $X_E(t,s,\xi )$ at time *t* of an individual, who had i-state $\xi$ at time *s*, equals *x*(*t*), where *x* is the solution to the initial value problem

2.6 $$\begin{aligned} \frac{d}{d \tau } x(\tau ) = g(x(\tau ),E(\tau )), \quad x(s)=\xi . \end{aligned}$$

We denote the rate at which individuals of i-state *x* produce children with i-state at birth in the measurable set $\omega$, when the environmental condition is *E*, by $\beta (x,E,\omega )$ and the death rate by $\mu (x,E)$. The probability that an individual, who had i-state $\xi$ at time *s*, is still alive at time *t*, given the course of the environmental condition (input), is

2.7 $$\begin{aligned} {\mathscr {F}}_E(t,s,\xi ) = e^{-\int _s^t \mu \left( X_E(\tau ,s,\xi ),E(\tau )\right) d\tau }. \end{aligned}$$

In terms of $\beta , \, X_E$ and ${\mathscr {F}}_E$ we have

2.8 $$\begin{aligned} \lambda _E(t,s)(\xi , \omega )= & {} \beta (X_E(t,s,\xi ),E(t),\omega ) {\mathscr {F}}_E(t,s,\xi ), \quad \omega \in {\mathscr {B}}(\varOmega _b), \end{aligned}$$

2.9 $$\begin{aligned} u_E(t,s,\xi )(\omega )= & {} \delta _{X_E(t,s,\xi )}(\omega ) {\mathscr {F}}_E(t,s,\xi ),\quad \omega \in {\mathscr {B}}(\varOmega ), \end{aligned}$$

and hence for deterministic i-state development (2.1) and (2.5) can be written as

2.10 $$\begin{aligned} b(t)(\omega )= & {} \int _{-\infty }^t \int _{\varOmega _b} \beta (X_E(t,s,\xi ),E(t),\omega ) {\mathscr {F}}_E(t,s,\xi ) b(s)(d\xi )ds,\quad \omega \in {\mathscr {B}}(\varOmega _b), \nonumber \\ \end{aligned}$$

2.11 $$\begin{aligned} m(t)(\omega )= & {} \int _{-\infty }^t \int _{\varOmega _b} \delta _{X_E(t,s,\xi )}(\omega ) {\mathscr {F}}_E(t,s,\xi )b(s)(d\xi )ds,\quad \omega \in {\mathscr {B}}(\varOmega ). \end{aligned}$$

If the environmental input is a given function of time, then all information is contained in the linear non-autonomous integral equation (2.10) for the birth rate *b*. But individuals affect their own environment, for instance by consuming food and exploiting other resources, in other words, there is feedback to the environment. This feedback mechanism is modelled by integro-differential equations prescribing how the histories of *b* and *E* affect the current rate of change of *E* (Diekmann et al. 2010).

## ODE reducibility

By a *population output* we mean a linear operator $O(E): M(\varOmega ) \rightarrow {\mathbb {R}}^k$ depending on the prevailing value *E* of the environmental input. Here we concentrate on outputs of the form

3.1 $$\begin{aligned} O(E)m = Q(E)N, \end{aligned}$$

where

3.2 $$\begin{aligned} N= \int _\varOmega w(\xi )m(d\xi ), \end{aligned}$$

with *w* a measurable function from $\varOmega$ to ${\mathbb {R}}^k$.

In many concrete models the output weight function depends on the environment and so one should replace *w* in (3.2) by ${\widetilde{w}}(\xi ,E)$. However, it turns out that then an ODE representation is possible only if

3.3 $$\begin{aligned} {\widetilde{w}}(\xi ,E) = Q(E)w(\xi ) \end{aligned}$$

which brings us back to (3.1) and (3.2).

If the population dynamics follows Eqs. (2.10) and (2.11), then the time evolution of the vector *N*(*t*) is given by

3.4 $$\begin{aligned} N(t) = \int _{-\infty }^t \int _{\varOmega _b} w(X_E(t,s,\xi )) {\mathscr {F}}_E(t,s,\xi )b(s)(d\xi )ds. \end{aligned}$$

We want to derive a condition for when the population output *N*(*t*) satisfies an ordinary differential equation

3.5 $$\begin{aligned} \frac{d}{dt}N(t) = K(E(t))N(t) \end{aligned}$$

for some $k \times k$-matrix valued function *K* of the environmental condition. Differentiating (3.4) and taking (2.10) into account, one finds that (3.5) holds if

3.6 $$\begin{aligned} (Dw(x)) g(x,E)-\mu (x,E)w(x) + \int _{\varOmega _b} w(\xi ) \beta (x,E,d\xi ) = K(E)w(x), \end{aligned}$$

where *Dw*(*x*) is the derivative of *w* at *x* represented by the $k \times q$ (Jacobian) matrix. In the case of one-dimensional i-state space $\varOmega$ ($q=1$), (3.6) of course simplifies to

3.7 $$\begin{aligned} g(x,E)w'(x) -\mu (x,E)w(x) + \int _{\varOmega _b} w(\xi ) \beta (x,E,d\xi ) = K(E)w(x). \end{aligned}$$

In most of the rest of the paper we restrict to the case $q=1$.

As explained in Sect. 2 one has, whenever feedback is present, to supplement the equation for the birth rate by an integro-differential equation describing the time evolution of the environment. Now, when we are looking for ODE reductions, this equation modelling the feedback should, of course, also be an ODE. If we know what properties of individuals are involved in the feedback, we know how to choose *w* and (3.7) becomes a condition on the model ingredients $g,\, \mu$ and $\beta$. It may very well happen that (3.7) does *not* hold if we take as components of *w* the functions that we really need to describe the feedback, but that (3.7) *does* hold if we add additional components (Diekmann et al., submitted, Example 1.2). A systematic procedure for finding such additional components is to compute the left-hand side of (3.7), eliminate everything that is a linear combination (with *E*-dependent coefficients) of the current components, see whether what remains can be written as a linear combination (again with *E*-dependent coefficients) of some new functions of *x* and then add these new functions as additional components. Now repeat. This procedure is described by Diekmann et al. (submitted) in more detail as a generally applicable test for ODE reducibility starting from a collection of output functionals that one needs or wants.

Alternatively one might try to characterise combinations of $g,\, \mu , \,\beta$ and *w* for which one can find *K*(*E*) such that (3.7) holds. In the paper (Diekmann et al., submitted) we restricted the submodel for reproduction by requiring that

3.8 $$\begin{aligned} \int _{\varOmega _b} w(\xi )\beta (x,E,d\xi ) = M(E)w(x) \end{aligned}$$

for some $k \times k$-matrix *M*(*E*). Note that this does indeed hold if $\beta$ has the form

3.9 $$\begin{aligned} \beta (x,E,\omega ) = \sum _{j=1}^{k} \beta _j(E,\omega )w_j(x), \end{aligned}$$

which motivates us to describe this situation as “reproduction is part of the output”. We then found a complete catalogue of combinations of $g,\, \mu$ and *w*, parametrised by *k* and various functions of either *x* or *E* (see $F_2$ and $F_3$ in Sect. 6).

In the present paper we extend the material of (Diekmann *et al.*, submitted) by providing some examples where (3.8) does not hold and by describing how large time ODE reduction is possible for models in which all physiological processes scale with the same factor depending on the environmental condition, so for a very strict form of physiological age.

## One-dimensional output and Monod’s model

When the population output is one-dimensional, that is, when *w* is a scalar function ($k=1$), the condition (3.7) says that the expression

4.1 $$\begin{aligned} \frac{g(x,E) w'(x) + \int _\varOmega w(\xi ) \beta (x,E,d\xi )}{w(x)} -\mu (x,E) \end{aligned}$$

should be independent of *x*.

Next we apply this result to the Monod model discussed in the introduction. To conform with the notation used there, we now denote the environmental condition by *S* and interpret it as substrate. The Monod model is formulated in terms of biomass *X* (which now plays the role of *N*), so

4.2 $$\begin{aligned} w(x) = x. \end{aligned}$$

Bacteria reproduce by fission into equal parts which we assume to happen at a rate $\beta _0(x,S)$ depending on the size *x* of the mother cell and the substrate concentration *S*. We further assume that fission is successful with probability *p*(*x*, *S*) in which case the two daughter cells are both half the size of their mother; otherwise the mother cell is lost without leaving any offspring. We neglect any other kind of mortality. These assumptions mean that the reproduction rate is given by

4.3 $$\begin{aligned} \beta (x,S,\omega ) = 2p(x,S)\beta _0(x,S) \delta _{\frac{x}{2}}(\omega ). \end{aligned}$$

With (4.2) and (4.3) we get

4.4 $$\begin{aligned} \int _\varOmega w(\xi ) \beta (x,S,d\xi ) = xp(x,S)\beta _0(x,S). \end{aligned}$$

According to our assumptions, cell loss happens at fission when the mother cell is lost at rate $\beta _0(x,S)$ and through washout at rate *D*. The total loss rate is the sum of these rates:

4.5 $$\begin{aligned} \mu (x,S) = \beta _0(x,S) +D. \end{aligned}$$

The condition for reducibility to the ODE (1.1) then becomes

4.6 $$\begin{aligned} \frac{g(x,S)}{x} - (1-p(x,S))\beta _0(x,S) -D = \frac{aS}{b+S} -D. \end{aligned}$$

In the case that every division is successful $p(x,S)=1$ and (4.6) becomes

4.7 $$\begin{aligned} g(x,S) = \frac{aS}{b+S} x, \end{aligned}$$

that is, individual growth rate per individual mass is exactly equal to the population growth rate per total biomass. The fission rate does not affect the finite dimensional representation in any way and can be chosen arbitrarily. When division may be unsuccessful, this type of mortality has to be compensated for in the individual growth rate and we have

4.8 $$\begin{aligned} g(x,S) = \left( \frac{aS}{b+S} + (1-p(x,S))\beta _0(x,S)\right) x. \end{aligned}$$

Note that in this case the individual growth rate and the fission rate are related.

## Two-dimensional output: a general formula and a particular example

We now describe how the previous class of examples can be extended. Our long term goal is to find a “maximal extension” in a similar manner as we did for the model class described in Sect. 6 below.

If $k=2$, one can interpret (3.7) as two linear equations in the two unknowns *g* and $\mu$. With the notational convention

5.1 $$\begin{aligned} \left\langle w,\beta \right\rangle = \int _{\varOmega _b} w(\xi )\beta (x,E,d\xi ) \end{aligned}$$

and while suppressing both arguments *x* and *E*, these equations are

5.2 $$\begin{aligned} \left( \begin{array}{cc} w_1' &{} -w_1 \\ w_2' &{} -w_2 \end{array} \right) \left( \begin{array}{c} g \\ \mu \end{array} \right) = \left( \begin{array}{c} k_{11}w_1 +k_{12}w_2 -\left\langle w_1,\beta \right\rangle \\ k_{21}w_1 +k_{22}w_2 -\left\langle w_2,\beta \right\rangle \end{array} \right) . \end{aligned}$$

We now solve (5.2) in a somewhat peculiar way in order to tie up with $F_3$ in Sect. 6. Since

5.3 $$\begin{aligned} \left( \begin{array}{cc} w_1' &{} -w_1 \\ w_2' &{} -w_2 \end{array} \right) ^{-1}= & {} \frac{1}{-w_1'w_2 +w_1w_2'} \left( \begin{array}{cc} -w_2 &{} w_1 \\ -w_2' &{} w_1' \end{array} \right) \nonumber \\= & {} \frac{1}{\left( \frac{w_2}{w_1}\right) '} \left( \begin{array}{cc} -\frac{w_2}{w_1}\frac{1}{w_1} &{} \frac{1}{w_1} \\ -\frac{w_1'}{w_1^2}\frac{w_2}{w_1} - \frac{1}{w_1}\left( \frac{w_2}{w_1}\right) ' &{} \frac{w_1'}{w_1^2} \end{array} \right) \end{aligned}$$

(note that $w_1$ and $w_2$ need to be linearly independent functions of the variable *x* so the Wronskian vanishes at most in isolated points), we obtain

5.4 $$\begin{aligned} g(x,E)= & {} \frac{1}{v'}\left( k_{21}+(k_{22}-k_{11})v-k_{12}v^2+v\frac{1}{w_1}\left\langle w_1,\beta \right\rangle - \frac{1}{w_1}\left\langle w_2,\beta \right\rangle \right) , \end{aligned}$$

5.5 $$\begin{aligned} \mu (x,E)= & {} \frac{w_1'}{w_1}g(x,E)-k_{11}-k_{12}v+\frac{1}{w_1}\left\langle w_1,\beta \right\rangle , \end{aligned}$$

where *v* is defined by

5.6 $$\begin{aligned} v := \frac{w_2}{w_1}. \end{aligned}$$

If reproduction is by division into two equal halves and if we incorporate the disappearance of the mother in $\beta$ rather than in $\mu$, we have

5.7 $$\begin{aligned} \left\langle w,\beta \right\rangle = \left( 2w\left( \frac{x}{2}\right) -w(x)\right) \beta _0(x,E). \end{aligned}$$

Next we choose

5.8 $$\begin{aligned} w_1(x) = x, \quad w_2(x)=x^{\frac{2}{3}}, \end{aligned}$$

so if we think of *x* as volume or mass, the $w_1$ measures volume and, assuming ball shaped organisms, $w_2$ measures surface area. So we want that substrate intake is proportional to $w_2$ and, ignoring metabolic costs, that *g* is proportional to substrate intake, hence to $w_2$.

From (5.8) we deduce

5.9 $$\begin{aligned} v(x) = x^{-\frac{1}{3}}, \quad v'(x)=-\frac{1}{3}x^{-\frac{4}{3}} \end{aligned}$$

and next from (5.4) and (5.5)

5.10 $$\begin{aligned} g(x,E)= & {} -3x^{\frac{4}{3}}\left( k_{21}+(k_{22}-k_{11}) x^{-\frac{1}{3}}-k_{12}x^{-\frac{2}{3}} -\frac{1}{x}\left( 2^{\frac{1}{3}}-1\right) x^{\frac{2}{3}} \beta _0(x,E)\right) ,\nonumber \\ \end{aligned}$$

5.11 $$\begin{aligned} \mu (x,E)= & {} \frac{1}{x}g(x,E)-k_{11}-k_{12}x^{-\frac{1}{3}}. \end{aligned}$$

We want both *g* and $\mu$ to be nonnegative (this is an aspect that received little attention in the paper (Diekmann et al., submitted)) and so there are some restrictions. The choice

5.12 $$\begin{aligned} \beta _0(x,E) = \frac{k_{21}}{2^{\frac{1}{3}}-1}x^{\frac{1}{3}}, \quad k_{11}=-D, \quad k_{22}=-D \end{aligned}$$

leads to

5.13 $$\begin{aligned} g(x,E) = 3k_{12}x^{\frac{2}{3}}, \quad \mu (x,E) = D+2k_{12}x^{-\frac{1}{3}} \end{aligned}$$

and from there to the ODE system

5.14 $$\begin{aligned} \frac{dN_1}{dt}= & {} -DN_1 + k_{12}(S)N_2, \end{aligned}$$

5.15 $$\begin{aligned} \frac{dN_2}{dt}= & {} k_{21}(S)N_1-DN_2. \end{aligned}$$

Assuming chemostat dynamics one complements Eqs. (5.14) and (5.15) with a third equation

5.16 $$\begin{aligned} \frac{dS}{dt} = D\left( S_\mathrm{ext} - S\right) - \gamma k_{12}(S)N_2, \end{aligned}$$

with the Michaelis–Menten uptake rate

5.17 $$\begin{aligned} k_{12}(S) =\frac{aS}{b+S} \end{aligned}$$

as a natural choice. From (5.12) $k_{21}(S)$ can be seen to represent the influence of the resource density on the size specific cell division rate. As yet little appears to be known about this. However, given the present technology, this function can in principle be determined experimentally. Moreover, one could attempt to determine it from some model of the cell cycle in dependence on *S*. (Another matter is whether in reality the division rate decomposes so neatly in a product of an *x*-dependent and a *S*-dependent term.) However, our best initial guess is to take $k_{21}$ to be constant.

## A catalogue of models that admit a finite dimensional state representation (a short recap of a long paper)

In Sect. 3 we already announced what is our best feat till now (Diekmann et al., submitted), a complete catalogue of all possible ODE reducible models for the case where the birth rate is treated as an output. Under this assumption we can in (3.6) drop the birth term from the backward operator as the births are already accounted for through the output map. Such models naturally allow an easy reconstruction of the full state trajectory. Additional technical assumptions were that the i-state space $\varOmega \subset {\mathbb {R}}$ is an interval (possibly of infinite length) and that $g(x,E_0)>0$ for all $x\in \varOmega$ for some constant environmental condition $E_0$. We then considered the problem

6.1 $$\begin{aligned} g(x,E)w'(x) -\mu (x,E)w(x) =H(E)w(x) \end{aligned}$$

with *w* taking values in ${\mathbb {R}}^k$ and *H* taking values in ${\mathbb {R}}^{k \times k}$.

Above we have already listed the condition that (4.1) should be independent of *x* if $k=1$ so we will not return to that case (represented by model family $F_1$ in the paper (Diekmann *et al.*, submitted)). For $k \ge 2$ we defined two families of functions *g* and $\mu$ for which we specified *w* and *H* such that (6.1) holds. These are presented in the following catalogue.

**Table:** 

$F_2$: Physiological age	
*k*	$k \ge 2$
Parameters	$\gamma _0:\varOmega \rightarrow {\mathbb {R}},\,\, \mu _0:{\mathscr {E}}\rightarrow {\mathbb {R}},\,\,v_0:\varOmega \rightarrow {\mathbb {R}},\,\,v_1:{\mathscr {E}}\rightarrow {\mathbb {R}}$
	$\varLambda \in {\mathbb {R}}^{k\times k}$ and $w(x_b) \in {\mathbb {R}}^k$
	$$\zeta (x):= \int _{x_b}^x \frac{dy}{v_0(y)}$$
*g*	$g(x,E) = v_0(x)v_1(E)$
$\mu$	$\mu (x,E) = \gamma _0(x)g(x,E) + \mu _0(E)$
*w*	$w(x) = \exp \left( \int _{x_b}^x \gamma _0(y)dy\right) \exp \left( \zeta (x)\varLambda \right) w(x_b)$
*H*	$H(E)=v_1(E)\varLambda -\mu _0(E)I$

and

**Table:** 

$F_3$: Generalised von Bertalanffy growth	
*k*	$k \ge 2$
Parameters	$\gamma _0:\varOmega \rightarrow {\mathbb {R}},\,\, \mu _0:{\mathscr {E}}\rightarrow {\mathbb {R}},\,\,v_0:\varOmega \rightarrow {\mathbb {R}},\,\,v_j:{\mathscr {E}}\rightarrow {\mathbb {R}},\,\, j=1,2,3.$
	$$\zeta (x):= \int _{x_b}^x \frac{dy}{v_0(y)}$$
*g*	$g(x,E) = v_0(x)\left( v_1(E)+v_2(E)\zeta (x)+v_3(E)\zeta (x)^2\right)$
$\mu$	$\mu (x,E) = \gamma _0(x)g(x,E) + \mu _0(E) +(k-1)v_3(E)\zeta (x)$
*w*	$w_j(x) = \exp \left( \int _{x_b}^x \gamma _0(y)dy\right) \zeta (x)^{j-1},\,\, j=1,2,\ldots ,k.$
*H*	$H(E)=H_0(E)-\mu _0(E)I$
	$$H_0=\left( \begin{array}{ccccc} 0 &{}-(k-1)v_3 &{}0 &{}\cdots &{}0\\ v_1 &{}v_2 &{}-(k-2)v_3 &{} &{}0 \\ 0 &{}2v_1 &{}2v_2 &{} &{}0\\ \vdots &{}0 &{}3v_1 &{} &{}0\\ &{} &{} &{} \ddots &{} \\ 0&{} \cdots &{} \cdots &{}(k-1)v_1 &{}(k-1)v_2\\ \end{array} \right) $$

Our main result is the following theorem.

### Theorem 6.1

Assume that $k \ge 2$ and that

(i) $g,\, \mu ,\,w$ and *H* are such that (6.1) holds,
(ii) $w_1,w_2,\ldots ,w_k$ are linearly independent functions of $x \in \varOmega$.

Then necessarily $g,\, \mu$ and *w* can be brought into the form specified in either $F_2$ or $F_3$ by a transformation of the i-state variable and a change of basis in ${\mathbb {R}}^k$.

In other words: The catalogue $F_2,\, F_3$ of solutions of (6.1) is complete.

The proof of Theorem 6.1 fills almost one half of the pages of the paper (Diekmann et al., submitted).

A few comments are in place. Firstly, it is useful to realise that a given function *g* may be of the form specified by $F_3$ even though, from the looks of it, one would not guess so. As a concrete example, consider

6.2 $$\begin{aligned} g(x,E)= v_1(E)(1+\cos x)+ v_2(E)\sin x + v_3(E)(1- \cos x). \end{aligned}$$

We challenge the reader to verify that, actually, the *g* given by (6.2) is of the form presented in $F_3$. Readers who do not like such a challenge are invited to consult Sect. 5 of the paper (Diekmann et al., submitted).

Secondly, in the derivation of the catalogue biological constraints such as positivity of the death rate have been ignored. However, afterwards one can check whether the various families in the catalogue contain cases that satisfy positivity and other biological constraints. One of the main purposes of the catalogue is that it can be used as background information at the modelling stage.

Finally, let us recall that the Eq. (6.1) and the families $F_2,\, F_3$ are concerned with a transport-degradation model without reproduction. They extend to full population models for which (3.9) holds. In addition they are relevant for models in which all components of *w* represent quantities that are conserved in the reproduction process, that is, models for which

6.3 $$\begin{aligned} \int _{\varOmega _b} w(\xi ) \beta (\xi ,E,d\xi ) = \beta _0(x,E)w(x) \end{aligned}$$

holds. To see this, simply observe that with the assumption (6.3) condition (3.7) reduces to (6.1) if one replaces $\mu$ by $\mu -\beta _0$.

## Physiological age and implicit scaling of time

In the developments till now we considered the requirements for a population model to be representable by an ODE whatever the initial condition. However not all applications require such a strong form of ODE representability. For example, when only some special initial conditions matter we can do with a weaker kind of ODE representability (which in analogy with similar problems in the theory of Markov chains (Kemeny and Snell 1960) we might call weak ODE representability). Note that such a special set should include also the subsequent trajectories and $\omega$-limit sets. A particularly important case is when we take the latter as our special initial conditions. We shall introduce this idea by means of an example inspired by Diekmann et al. (1983) and Metz and Diekmann (1986, I.4.5, II.14). Our aim is to explain how the ideas presented there fit in with ODE reducibility as discussed above. The first step is simple. Suppose that

7.1 $$\begin{aligned}&g(x,E) = \vartheta (E)g_0(x), \end{aligned}$$

7.2 $$\begin{aligned}&\mu (x,E) = D+\vartheta (E)\mu _0(x), \end{aligned}$$

7.3 $$\begin{aligned}&\beta (x,E,\omega ) =\vartheta (E)\beta _0(x,\omega ). \end{aligned}$$

Then (4.1) is independent of *x* if

7.4 $$\begin{aligned} g_0(x)w'(x) -\mu _0(x)w(x) +\int _{\varOmega _b} w(\xi )\beta _0(x,d\xi ) = \lambda _dw(x) \end{aligned}$$

holds for some real number $\lambda _d$. We have suggestively provided $\lambda$ with a subscript *d* for “dominant”. Correspondingly we assume that *w* is nonnegative. There exists a large literature to support this, see for instance Heijmans (1986a, b) and Bátkai et al. (2017) for general theory and the wider context. With this choice of *w* we find for *N* defined by (3.4) the ODE

7.5 $$\begin{aligned} \frac{dN}{dt} = -DN +\lambda _d\vartheta (E)N. \end{aligned}$$

So far so good. The difficulty is, of course, that (7.4) provides an implicit characterisation of *w* and that we do not have any form of control. So, in general, this *w* is not suitable as a block for building a community model. Yet, as explained in detail in Diekmann (1983), Metz and Diekmann (1986, I.4.5, II.14), the variable *N* can be used to describe the asymptotic large time behaviour even if it fails to capture all relevant aspects of the transient phase. A crucial observation in this context is that the change of time variable

7.6 $$\begin{aligned} \frac{d\tau }{dt} = \vartheta (E(t)), \quad \tau (0) =0 \end{aligned}$$

from *t* to $\tau$ makes the problem linear, enabling us to use the theory of linear positive semigroups even though the original problem is nonlinear. The main result is that the large time behaviour of *N* and $E=S$ is described by the two-dimensional system consisting of (7.5) and

7.7 $$\begin{aligned} \frac{dS}{dt} = D\left( S_\mathrm{ext}-S\right) -\gamma \vartheta (S)N. \end{aligned}$$

This type of asymptotic ODE reducibility was already described explicitly by Val and Metz (unpublished). They additionally considered a class of models with higher dimensional i-state space where ODE reducibility holds for an invariant subset of the population state space (while providing numerical evidence that the subset is an attractor).

## Discussion

Physiologically structured population models have the advantage that one can incorporate mechanistic detail of i-level behaviour. Thus it becomes possible to investigate the relationship between mechanisms at the i-level and phenomena at the p-level (Diekmann et al. 2010; de Roos and Persson 2013). But physiologically structured population models also have disadvantages. There are only few numerical tools for their study, see Brännström et al. (2013), Carrillo et al. (2014), Breda et al. (2016), Aye and Carlsson (2017), Zhang et al. (2017), de Roos (2018) and Scarabel (2018). In this respect the situation for population models formulated in terms of ODEs is infinitely better. Moreover, there is a highly developed qualitative theory for ODEs that only partly carries over to infinite dimensional dynamical systems and when it does, answering questions concerning for instance the structure of $\omega$-limit sets may be exceedingly hard. It is therefore of interest to know when a physiologically structured model can be reduced to an ODE and when it cannot.

Structured population models involve book-keeping considerations and there are multiple ways to organise these. Accordingly, there are as well multiple ways of associating a dynamical system at the population level with the model (Barril et al in preparation; Calsina et al. 2016). We favour book-keeping based on an individual’s state-at-birth and age, which leads to an abstract renewal equation for the population level birth rate (Diekmann and Gyllenberg 2007). This is particularly helpful when there is only one state-at-birth in which case the renewal equation becomes a scalar one (Diekmann et al. 2010).

In this paper we showed how the renewal equation leads, in just a few lines, to the condition (3.6) for ODE reducibility. The version (3.7) involves the per capita growth, death, and reproduction rates $g,\, \mu$ and $\beta$ as well as an ${\mathbb {R}}^k$ valued function *w* of per capita outputs. After paying special attention to the cases $k=1$ and $k=2$, in particular for fission into two equal parts, we reviewed the catalogue derived by Diekmann et al (submitted) and shown by them to be complete under certain restrictions. In addition we revealed the connection between (3.7) and the example of Diekmann (1983), Metz and Diekmann (1986, I.4.5, II.14) allowing reduction of large time behaviour to an ODE.

Originally we had some hope of being able to derive results (perhaps only partial) for the rather special, but also important, case

8.1 $$\begin{aligned} \beta (x,E,\omega ) = \beta _0(x,E)\delta _{x_b}(\omega ) \end{aligned}$$

of only one possible state-at-birth $x_b$. In fact we got nowhere. So we like to present this as an open problem.
